# Explanatory role of sociodemographic, clinical, behavioral, and social factors on cognitive decline in older adults with diabetes

**DOI:** 10.1186/s12877-021-02740-7

**Published:** 2022-01-10

**Authors:** Sean M. O’Toole, Rebekah J. Walker, Emma Garacci, Aprill Z. Dawson, Jennifer A. Campbell, Leonard E. Egede

**Affiliations:** 1 College of Medicine, Medical College of Wisconsin, Milwaukee, WI USA; 2 Division of General Internal Medicine, Department of Medicine, Medical College of Wisconsin, Milwaukee, WI USA; 3 Center for Advancing Population Science (CAPS), Medical College of Wisconsin, Milwaukee, WI USA

**Keywords:** Cognitive decline, health disparities, elderly, diabetes, Health and Retirement Survey

## Abstract

**Background:**

The aim of the study was to examine the explanatory role of sociodemographic, clinical, behavioral, and social factors on racial/ethnic differences in cognitive decline among adults with diabetes.

**Methods:**

Adults aged 50+ years with diabetes from the Health and Retirement Survey were assessed for cognitive function (normal, mild cognitive impairment [MCI], and dementia). Generalized estimating equation (GEE) logistic regression models were used to account for repeating measures over time. Models were adjusted for sociodemographic (gender, age, education, household income and assets), behavioral (smoking), clinical (ie. comorbidities, body mass index), and social (social support, loneliness, social participation, perceived constraints and perceived mastery on personal control) factors.

**Results:**

Unadjusted models showed non-Hispanic Blacks (NHB) and Hispanics were significantly more likely to progress from normal cognition to dementia (NHB OR: 2.99, 95%CI 2.35–3.81; Hispanic OR: 3.55, 95%CI 2.77–4.56), and normal cognition to MCI (NHB OR = 2.45, 95%CI 2.14–2.82; Hispanic OR = 2.49, 95%CI 2.13–2.90) compared to non-Hispanic Whites (NHW). Unadjusted models for the transition from mild cognitive decline to dementia showed Hispanics were more likely than NHW to progress (OR = 1.43, 95%CI 1.11–1.84).

After adjusting for sociodemographic, clinical/behavioral, and social measures, NHB were 3.75 times more likely (95%CI 2.52–5.56) than NHW to reach dementia from normal cognition. NHB were 2.87 times more likely (95%CI 2.37–3.48) than NHW to reach MCI from normal. Hispanics were 1.72 times more likely (95%CI 1.17–2.52) than NHW to reach dementia from MCI.

**Conclusion:**

Clinical/behavioral and social factors did not explain racial/ethnic disparities. Racial/ethnic disparities are less evident from MCI to dementia, emphasizing preventative measures/interventions before cognitive impairment onset are important.

## Introduction

Diabetes is one of the most common chronic diseases, affecting roughly 9.4% of the U.S. population [1]. Racial/ethnic minorities have higher rates of diagnosed diabetes than non-Hispanic Whites, which increases risks of developing comorbidities such as cardiovascular disease, stroke, and kidney disease [1]. Additionally, diabetes has been associated with increased decline in cognitive functioning [2–5].

Cognitive decline encompasses a spectrum from normal cognition to mild cognitive impairment (MCI), followed by dementia – with mild cognitive impairment (MCI) a recognized intermediate condition between age-appropriate declines in cognition and diagnosable dementia [5–8]. Diabetes may increase one’s risk for the progression from MCI to dementia, in addition to the increased risk of progression from normal cognition to dementia [9, 10]. While not characteristic for every individual with diabetes, cognitive deficits that may be exhibited include slower mental and motor processing, executive function and attention, and diminishments in learning and memory, which affect a patient’s management of their diabetes, and their overall quality of life [4, 11–13]. Cognitive deficits in older adults with diabetes show profound differences across different races and ethnicities; for example, non-Hispanic Blacks, Hispanics, Native Americans, and Mexican Americans have greater cognitive deficits over time compared to non-Hispanic Whites (NHW) [14–20].

Efforts to explain these differences across race and ethnicity suggest social determinants of health, such as socioeconomic status, comorbidities, social support, and loneliness are potential contributors [14–23]. Participation in mental, social, and physical leisure activities, along with having a strong social network, have also been recently indicated as possible protective factors against cognitive decline [23]. While the influence of social determinants of health on racial/ethnic disparities [22] and on outcomes in individuals with diabetes, such as glycemic control and quality of life, have been indicated [24], whether social determinants of health help explain racial/ethnic disparities in cognitive decline in older adult populations has not been investigated. In addition, current literature on racial/ethnic disparities in diabetes-related cognitive decline have not investigated the intermediate stage of MCI to determine whether these racial/ethnic disparities are more pronounced at different stages along the cognitive spectrum.

The aim of this paper was to investigate the explanatory role of sociodemographic, clinical, behavioral, and social factors on racial/ethnic differences in cognitive decline among older adults with diabetes. Analyses were planned to expand upon existing work by investigating the intermediate stage of cognitive decline and dementia. In addition, covariates were included to assess whether participation in a social network and leisure activities explained racial differences in cognitive decline amongst older adults with diabetes. We hypothesized that after accounting for differences in sociodemographic, clinical, behavioral, and social factors, the disparities by race/ethnicity would diminish.

## Methods

### Data Source and Study Population

The Health and Retirement Study (HRS) is a nationally representative, longitudinal survey of U.S. adults over age 50 and their spouses [25]. The primary goal of HRS is to examine ways in which the health of older adults interacts with social, economic, and psychological factors and retirement decisions. Respondents are interviewed every two years on their financial, health and family status. Biennial interviews were conducted through 2014 and include the enhanced face-to-face (EFTF) interview that collects a set of physical performance measures, cognitive performance tests and biomarkers, and a Leave-Behind Questionnaire that collects questionnaires on social factors, such as perceived social support, loneliness, social participation, and personal control. A random one-half of households were pre-selected for the EFTF in 2006, with the other half of the sample selected for 2008, and from that point on every household will repeat the EFTF portion every other wave [25].

Inclusion in this study required 1) participants completed the Leave-Behind Questionnaire so each individual would have data for all factors investigated in final models, 2) completion of the cognitive test allowing outcome measurement for each individual, and 3) self-report of diabetes defined by answering ‘yes’ to the question, “has a doctor ever told you that you have diabetes or high blood sugar?”. Participants were excluded if they did not self-report race/ethnicity to allow investigation into racial/ethnic disparities across all participants. In total, 22,295 participants were eligible for the Leave-Behind Questionnaire from 2008 to 2014. Among them, 21,356 participants were ages 50 years and older during the first cognitive test in the time period, allowing collection of cognitive function information. Of these, 5576 participants self-reported having diabetes. After excluding 5 without race/ethnicity reported, 5571 participants were included in the sample. There was a total of 8399 cognitive performance tests for selected participants completed between 2008 and 2014 [24].

### Outcome

The outcome for this study was cognitive function, categorized as normal, mild cognitive impairment, and dementia. The cognitive functioning measures within HRS include: immediate word recall (score: 0–10), delayed word recall (score: 0–10), serial 7 s (score: 0–5), and backwards count from 20 (score: 0–2). Combining these scores, HRS provides a total cognitive function score for each individual that ranges from 0–27. This was categorized into dementia (score: 0–6), mild impairment (score: 7–11), and normal cognition (score: 12–27) based on standard cut-points [14, 26]. Cognitive measures were pulled from the RAND HRS Longitudinal file 2014 (V2). Values for missing measures were imputed by HRS prior to data release using a multivariate, regression-based procedure using Imputation and Variance Estimation (IVEware) software [27].

### Predictors and covariates

#### Race/Ethnicity

The primary predictor was self-reported race/ethnicity, and was categorized as non-Hispanic White, non-Hispanic Black, Hispanic, and Other minority. This variable was created from two original questions, one asking the race of the individual with options of ‘White/Caucasian’, ‘Black/African American’, and ‘Other’; and a second question asking ethnicity with options of ‘Not Hispanic’ and ‘Hispanic’. Individuals responding Hispanic to the ethnicity question were categorized as Hispanic regardless of race selection. Individuals selecting White and Not Hispanic were categorized as non-Hispanic White, those selecting Black and Not Hispanic were categorized as non-Hispanic Black, and those selecting Other and Not Hispanic were categorized as Other minority.

#### Sociodemographic Factors

Demographic and socioeconomic factors included gender (male/female), age (in years), education (less than high school, GED/high school graduate, some college, college and above), household income and assets which included shares of stock, jewelry, and real estate (grouped into quartiles). Sociodemographic factors were pulled from the RAND HRS data [28].

#### Clinical/Behavioral Factors

Behavioral factors included smoking status (grouped as non-smoker, current smoker, and former smoker). Comorbidities included a count of self-reported conditions: high blood pressure, cancer, lung disease, heart condition, stroke, emotional/psychiatric problems, and arthritis. Comorbidities were then grouped as low comorbidity (0–1), moderate comorbidity (2–3), and high comorbidity (4+). BMI was categorized as underweight, normal weight, overweight, and obese. Behavioral factors and comorbidities were pulled from the RAND HRS data [28].

#### Social Factors

Social factors included social support (positive social support score and negative social support score), loneliness (a score of the original 3-item loneliness index); social participation (a score of 18 items), personal sense of control (separated into perceived constraints score and perceived mastery score) [29]. Social support was based on 1–4 scale from four sets of 7 items, which examined the perceived support that respondents received from their spouse/partner, children, family, and friends. For each relationship category there are 3 positively worded items and 4 negatively worded items. Positive and negative social support scores are determined by averaging the reverse-coded scores within each dimension with higher scores for positive social support indicating higher levels of positive social support, and higher scores for negative social support indicating higher levels of negative social support. Loneliness was based on 1–3 scale, which asks questions such as “do you lack companionship?”, “feel left out?”, “feel isolated from others?”. Scores are determined by averaging the scores across all 3 reverse- coding items, with higher scores indicating higher levels of loneliness. Social participation was based on 0–18 scale from 18 items. For each item, an indicator was created, with 1 indicating yes for answers with daily, several times a week, once a week, several times a month, at least once a month; 0 for answers with not in the last month, never/not relevant. Scores were determined by summing the indicator of each item, with higher scores indicating higher levels of social participation. Personal sense of control was based on 1–6 scale from two sets of 5 items, 5 items for constraints and 5 items for mastery. The final scores were constructed by averaging the scores across 5 items, with higher scores indicating higher levels of personal sense of control. Social factors were pulled from the biennial core interview data and scoring was based on HRS documentation [29].

### Statistical Analysis

To analyze whether race/ethnicity was associated with cognitive decline, we fit a series of generalized estimating equation (GEE) logistic regression models. The GEE approach was used to control for non-independence among the repeated observations for each individual. Then, a series of models were fit to determine if sociodemographic, clinical/behavioral, or social factors explained racial/ethnic differences in cognitive decline. All *p*-values were 2-sided and *p* < .05 was considered statistically significant. Statistical analysis was performed with SAS version 9.4 (SAS Institute).

First, a series of univariate analyses using ANOVA were conducted to investigate sample characteristic differences by cognitive function (normal cognition, mild cognitive impairment, dementia). Second, three sets of unadjusted GEE logistic models were fit with race/ethnicity as the primary independent variable. The cognitive decline outcome was modeled as 1) dementia vs. normal (reference); 2) mild cognitive decline vs. normal (reference); 3) dementia vs. mild cognitive decline (reference). Finally, three sets of adjusted GEE logistic models were run adding in variables in blocks that included sociodemographic factors, clinical/behavioral factors, and social factors. Models were fit by adding the three groups of factors one by one, followed by a fully adjusted model that included all covariates. Therefore, Adjusted Model 1 (Sociodemographics) included Gender, Age, Education, Household Income/Assets. Adjusted Model 2 (Clinical/Behavioral) included number of Comorbidities, BMI, Smoking Status. Adjusted Model 3 (Social) included Perceived Support Loneliness, Social Participation, Perceived Constraints on Personal Control, Perceived Mastery on Personal Control. The Fully Adjusted Model included all Sociodemographic, Clinical/Behavioral, and Social factors.

## Results

Table 1 presents sample characteristics stratified by cognitive function. The sample included 5571 older adults with diabetes. Based on cognitive function status at the time of first report: 71.9% (*n* = 4007) were of ‘normal function’, 22.5% (*n* = 1255) ‘mild cognitive impairment’, and 5.6% (*n* = 309) ‘dementia’ status. 54.9% (*n* = 3060) of the sample were Non-Hispanic White (NHW), 24.1% (*n* = 1340) Non-Hispanic Black (NHB), 17.3% (*n* = 962) Hispanic, and 3.8% (*n* = 209) ‘Other’. 55.3% (*n* = 3082) were female, and the mean age was 67 years old.

**Table 1:** Sample characteristics by cognitive status

	Total (***n*** = 5571)	Normal (***n*** = 4007)	Mild (***n*** = 1255)	Dementia (***n*** = 309)	***P***-value
Demographics					
**Race/Ethnicity**					<.0001
Non-Hispanic White (NHW)	54.9%	61.2%	39.8%	34.6%	
Non-Hispanic Black (NHB)	24.1%	20.5%	33.1%	33.7%	
Hispanic	17.3%	14.3%	23.9%	28.8%	
Other	3.8%	4.0%	3.2%	2.9%	
**Gender**					0.0002
Male	44.7%	46.4%	41.0%	37.9%	
Female	55.3%	53.6%	59.0%	62.1%	
**Age at first assessment**					<.0001
Mean (SD)	67.2 (9.88)	66.0 (9.26)	69.6 (10.57)	73.5 (10.71)	
**Education level**					<.0001
Less than high school	26.3%	17.4%	45.3%	65.1%	
GED/High school graduate	34.5%	35.7%	33.0%	24.6%	
Some college	22.9%	26.6%	15.0%	7.1%	
College and above	16.3%	20.3%	6.8%	3.2%	
**Household income and assets [median (IQR)/quartile]**					
1st Quartile	$18,312 (8906–36,512)	$20,412 (9292–39,096)	$16,729 (8764–34,480)	$12,000 (8088–25,008)	0.58
2nd Quartile	$122,896 (88,618 - 160,250)	$125,300 (90,224 - 162,000)	$116,596 (83,432 - 153,200)	$128,148 (79,780 - 160,799)	0.03
3rd Quartile	$313,010 (250,537 - 410,561)	$314,028 (250,412 - 410,000)	$308,697 (249,000 - 419,500)	$317,052 (272,902 - 412,359)	0.68
4th Quartile	$888,267 (680,020 - 1,388,797)	$899,800 (678,888 - 1,411,640)	$847,865 (700,100 - 1,113,872)	$833,672 (637,253 - 1,846,832)	0.48
Clinical/Behavioral					
**Comorbidity group**					<.0001
Low comorbidity (0–1)	25.9%	28.1%	21.3%	16.8%	
Moderate comorbidity (2–3)	53.1%	53.1%	53.5%	51.8%	
High comorbidity (4+)	21.0%	18.9%	25.3%	31.4%	
**BMI category**					<.0001
Underweight	0.6%	0.3%	1.1%	2.0%	
Normal weight	14.5%	12.8%	17.9%	22.9%	
Overweight	32.7%	32.0%	34.2%	36.6%	
Obese	52.2%	54.9%	46.8%	38.5%	
**Smoking status**					0.07
Non-smoker	41.5%	42.3%	38.3%	44.3%	
Former smoker	45.1%	44.9%	46.7%	42.4%	
Current smoker	13.4%	12.8%	15.0%	13.3%	
Social Factors					
**Perceived social support –positive**					0.99
Mean (SD)	3.09 (0.57)	3.09 (0.55)	3.09 (0.61)	3.09 (0.64)	
**Perceived social support –negative**					0.48
Mean (SD)	1.71 (0.51)	1.70 (0.49)	1.72 (0.57)	1.69 (0.55)	
**Loneliness**					<.0001
Mean (SD)	1.55 (0.57)	1.53 (0.56)	1.62 (0.58)	1.64 (0.59)	
**Social participation**					<.0001
Mean (SD)	7.38 (3.29)	7.91 (3.12)	6.15 (3.21)	4.61 (3.36)	
**Perceived constraints on personal control**					<.0001
Mean (SD)	2.38 (1.25)	2.25 (1.19)	2.71 (1.34)	2.98 (1.34)	
**Perceived mastery on personal control**					<.0001
Mean (SD)	4.65 (1.16)	4.72 (1.11)	4.49 (1.25)	4.14 (1.40)	

*SD* standard deviation

Table 2 presents sample characteristics stratified by race/ethnicity. At the time of the first assessment, 80.2% (*n* = 2453) of NHW were characterized as having ‘normal cognitive function’, 16.3% (*n* = 500) ‘mild’, and 3.5% (*n* = 107) ‘dementia’. 61.3% (*n* = 821) of NHB were of ‘normal’ cognition, 31.0% (*n* = 415) ‘mild’, and 7.7% (*n* = 104) ‘dementia’. 59.6% (*n* = 573) of Hispanics were of ‘normal’ cognition, 31.2% (*n* = 300) ‘mild’, and 9.2% (*n* = 89) ‘dementia’. 76.6% (*n* = 160) of Other minorities were of ‘normal’ cognition, 19.1% (*n* = 40) ‘mild’, and 4.3% (*n* = 9) ‘dementia’.

**Table 2:** Sample characteristics by race/ethnicity

	Total (***n*** = 5571)	NH White (***n*** = 3060)	NH Black (***n*** = 1340)	Hispanic (***n*** = 962)	Other (***n*** = 209)	***P***-value
Demographics						
**Cognitive Status**						<.0001
Normal	71.9%	80.2%	61.3%	59.6%	76.6%	
Mild	22.5%	16.3%	31.0%	31.2%	19.1%	
Dementia	5.6%	3.5%	7.7%	9.2%	4.3%	
**Gender**						<.0001
Male	44.7%	48.7%	37.6%	41.3%	46.4%	
Female	55.3%	51.3%	62.4%	58.7%	53.6%	
**Age in years at first assessment**						<.0001
Mean (SD)	67.2 (9.88)	69.5 (9.87)	64.8 (9.11)	64.4 (9.34)	62.5 (8.42)	
**Education level**						<.0001
Less than high school	26.3%	15.8%	28.7%	58.3%	17.2%	
GED/High school graduate	34.5%	39.7%	33.2%	20.9%	28.2%	
Some college	22.9%	24.6%	25.0%	14.8%	22.5%	
College and above	16.3%	19.9%	13.1%	6.0%	32.1%	
**Household income and assets [median (IQR)/quartile]**						
1st Quartile	$18,312 (8906–36,512)	$23,528 (11,052 - 40,016)	$15,142 (8149–34,000)	$15,900 (8217–33,677)	$19,108 (8020–42,513)	0.03
2nd Quartile	$122,896 (88,618 - 160,250)	$128,532 (97,224 - 166,212)	$116,285 (83,216 - 154,240)	$112,122 (79,200 - 156,000)	$98,801 (80,008 - 134,370)	<.0001
3rd Quartile	$313,010 (250,537 - 410,561)	$322,872 (252,056 - 420,460)	$295,608 (243,610 - 379,432)	$302,900 (254,882 - 402,610)	$283,824 (242,354 - 411,179)	0.09
4th Quartile	$888,267 (680,020 - 1,388,797)	$931,802 (702,836 - 1,423,416)	$756,421 (624,170 - 1,170,000)	$702,000 (622,060 - 985,000)	$843,029 (683,492 - 1,337,488)	0.03
Clinical/Behavioral						
**Comorbidity group**						<.0001
Low comorbidity (0–1)	25.9%	21.4%	24.9%	38.6%	40.7%	
Moderate comorbidity (2–3)	53.1%	54.7%	54.7%	47.9%	42.6%	
High comorbidity (4+)	21.0%	23.9%	20.4%	13.5%	16.7%	
**BMI category**						0.001
Underweight	0.6%	0.5%	0.6%	0.9%	0.5%	
Normal weight	14.5%	15.0%	13.4%	13.2%	19.6%	
Overweight	32.7%	34.1%	28.7%	33.1%	35.8%	
Obese	52.2%	50.4%	57.3%	52.8%	44.1%	
**Smoking status**						<.0001
Non-smoker	41.5%	40.7%	38.4%	47.1%	46.4%	
Former smoker	45.1%	47.5%	43.8%	41.1%	37.8%	
Current smoker	13.4%	11.8%	17.8%	11.8%	15.8%	
Social						
**Perceived social support – positive**						0.42
Mean (SD)	3.09 (0.57)	3.08 (0.55)	3.11 (0.59)	3.11 (0.60)	3.07 (0.56)	
**Perceived social support – negative**						<.0001
Mean (SD)	1.71 (0.51)	1.65 (0.47)	1.81 (0.56)	1.76 (0.55)	1.90 (0.57)	
**Loneliness**						<.0001
Mean (SD)	1.55 (0.57)	1.53 (0.57)	1.63 (0.58)	1.52 (0.56)	1.60 (0.60)	
**Social participation**						<.0001
Mean (SD)	7.38 (3.29)	7.61 (3.21)	7.27 (3.58)	6.50 (3.07)	7.92 (2.96)	
**Perceived constraints on personal control**						<.0001
Mean (SD)	2.38 (1.25)	2.34 (1.23)	2.34 (1.20)	2.59 (1.35)	2.54 (1.36)	
**Perceived mastery on personal control**						0.02
Mean (SD)	4.65 (1.16)	4.65 (1.11)	4.56 (1.25)	4.71 (1.24)	4.82 (1.14)	

*NH Non-Hispanic

**SD Standard Deviation

Table 3 shows the results of the GEE models examining the relationship between race/ethnicity and cognitive function. In unadjusted models, compared to NHW, NHB and Hispanics were significantly more likely to progress from normal cognition to dementia (NHB OR: 2.99, 95%CI 2.35–3.81; Hispanic OR: 3.55, 95%CI 2.77–4.56). This significance remained despite adjustment for sociodemographic, clinical/behavioral, or social factors. In fully adjusted models NHB were still 3.75 times more likely, and Hispanics 3.05 times more likely to progress from normal cognitive functioning to dementia (NHB OR = 3.75, 95% CI 2.52–5.56; Hispanic OR = 3.05, 95% CI 1.97–4.72). In addition, Other minorities were 3.82 times more likely to progress from normal cognition to dementia (OR = 3.82, 95%CI 1.47–9.95). While the strength of association was somewhat weakened for Hispanics in the fully adjusted model, it was not for NHB or Other minorities.

**Table 3:** Relationship between race/ethnicity and cognitive function

**Dementia vs. Normal (ref)**										
	**Unadjusted**		**Adjusted Model 1**		**Adjusted Model 2**		**Adjusted Model 3**		**Fully Adjusted**	
	*OR (95% CI)*	*P-value*	*OR (95% CI)*	*P-value*	*OR (95% CI)*	*P-value*	*OR (95% CI)*	*P-value*	*OR (95% CI)*	*P-value*
**Race/Ethnicity**		<.0001		<.0001		<.0001		<.0001		<.0001
NH White	Ref		Ref		Ref		Ref		Ref	
NH Black	2.99 (2.35–3.81)		3.36 (2.51–4.50)		3.33 (2.59–4.28)		3.35 (2.43–4.63)		3.75 (2.52–5.56)	
Hispanic	3.55 (2.77–4.56)		2.23 (1.61–3.11)		4.24 (3.25–5.53)		3.52 (2.52–4.93)		3.05 (1.97–4.72)	
Other	1.09 (0.56–2.11)		2.33 (1.13–4.80)		1.12 (0.57–2.20)		1.73 (0.79–3.78)		3.82 (1.47–9.95)	
**Mild Cognitive Impairment vs. Normal (ref)**										
	**Unadjusted**		**Adjusted Model 1**		**Adjusted Model 2**		**Adjusted Model 3**		**Fully Adjusted**	
	*OR (95% CI)*	*P-value*	*OR (95% CI)*	*P-value*	*OR (95% CI)*	*P-value*	*OR (95% CI)*	*P-value*	*OR (95% CI)*	*P-value*
**Race/Ethnicity**		<.0001		<.0001		<.0001		<.0001		<.0001
NH White	Ref		Ref		Ref		Ref		Ref	
NH Black	2.45 (2.14–2.82)		2.64 (2.25–3.09)		2.65 (2.30–3.06)		2.63 (2.22–3.11)		2.87 (2.37–3.48)	
Hispanic	2.49 (2.13–2.90)		1.84 (1.53–2.22)		2.75 (2.34–3.23)		2.16 (1.78–2.62)		1.88 (1.49–2.37)	
Other	1.23 (0.88–1.73)		1.83 (1.28–2.61)		1.30 (0.91–1.86)		1.49 (1.00–2.23)		2.20 (1.42–3.39)	
**Dementia vs. Mild Cognitive Impairment (ref)**										
	**Unadjusted**		**Adjusted Model 1**		**Adjusted Model 2**		**Adjusted Model 3**		**Fully Adjusted**	
	*OR (95% CI)*	*P-value*	*OR (95% CI)*	*P-value*	*OR (95% CI)*	*P-value*	*OR (95% CI)*	*P-value*	*OR (95% CI)*	*P-value*
**Race/Ethnicity**		0.04		0.28		0.01		0.01		0.04
NH White	Ref		Ref		Ref		Ref		Ref	
NH Black	1.22 (0.95–1.56)		1.29 (0.98–1.69)		1.32 (1.02–1.70)		1.38 (1.00–1.90)		1.44 (1.00–2.06)	
Hispanic	1.43 (1.11–1.84)		1.27 (0.94–1.70)		1.59 (1.22–2.07)		1.74 (1.26–2.39)		1.72 (1.17–2.52)	
Other	0.88 (0.44–1.77)		1.17 (0.56–2.44)		1.00 (0.50–2.03)		1.26 (0.55–2.89)		1.72 (0.68–4.31)	

*Adjusted Model 1: **Sociodemographics** *(Gender, Age, Education, Household Income/Assets)*

**Adjusted Model 2: **Clinical/Behavioral** *(# of Comorbidities, BMI, Smoking Status)*

***Adjusted Model 3: **Social** *(Perceived Support (+), Perceived Support (−), Loneliness, Social Participation, Perceived Constraints on Personal Control, Perceived Mastery on Personal Control)*

****Fully Adjusted Model: **Demographics** + **Clinical/Behavioral** + **Social**

In the progression from normal cognitive functioning to mild cognitive impairment, unadjusted models showed significant differences for NHB and Hispanics compared to NHW, but odds ratios were lower than progression from normal cognition to dementia (NHB OR = 2.45, 95%CI 2.14–2.82; Hispanic OR = 2.49, 95%CI 2.13–2.90). Again, this significance held despite adjustment, and in the fully-adjusted model NHB were 2.87 times more likely (OR = 2.87, 95% CI 2.37–3.48) and Hispanics were 1.88 times more likely than NHW to progress from normal cognition to mild impairment. In addition, Other minorities were 2.20 times more likely to progress from normal cognition to mild impairment (OR = 2.20, 95%CI 1.42–3.39). Similar to progression from normal cognition to dementia, while the strength of association was somewhat weakened for Hispanics in the fully adjusted model, it was not for NHB or Other minorities.

For the transition from mild cognitive decline to dementia, in unadjusted models, Hispanics were significantly more likely than NHW to progress (OR = 1.43, 95%CI 1.11–1.84). While adjustment for sociodemographic factors removed all significance, adjustment for clinical/behavioral and social factors strengthened the relationship between race/ethnicity and progression. In the fully-adjusted model that incorporated all covariates, NHB were 1.44 times more likely and Hispanics were 1.72 times more likely to progress from mild cognitive impairment to dementia compared to NHW (NHB OR = 1.44, 95% CI 1.00–2.06; Hispanics OR = 1.72, 95% CI 1.17–2.52). This was the only progression where Hispanics had a stronger relationship than NHB compared to NHW, and the relationship was not weakened after adjustment.

## Discussion

This study examined differences in cognitive function by race and ethnicity among individuals with diabetes using the national longitudinal Health and Retirement Study (HRS). A systematic approach was taken to assess demographic, clinical/behavioral, and social factors, first individually and then comprehensively, to explain the disparities across race/ethnicity in diabetes-related cognitive decline. Contrary to our hypothesis, there was minimal change in the odds ratios after adjustment, suggesting social factors previously hypothesized to explain disparities did not explain differences by race/ethnicity. By examining progression across three phases of cognitive decline, this study also found that the overall strength of racial/ethnic differences was higher in the progression from normal to mild cognitive decline (as noted by comparatively higher odds ratios), suggesting the importance of understanding drivers of differences in diabetes-related cognitive decline at early stages to minimize further exacerbation of disparities.

This study adds to the current literature by providing a broader investigation across the spectrum of cognition, and a wide range of possible explanatory factors to guide interventions targeting racial/ethnic disparities. Previous studies primarily focused on the relationship between race/ethnicity and dementia among older patients with diabetes [15, 17, 19]. Consistent with previous studies there was a significant difference in cognitive decline by race/ethnicity, with minorities having a higher likelihood of progression to both mild cognitive decline and dementia [14–20]. By including mild cognitive impairment, this study provides additional information by identifying differences that exist between race/ethnicity during the progression from normal to mild cognitive decline may be stronger and therefore are an important stage during which to focus efforts aimed at decreasing disparities. In addition, while social factors have been noted in prior studies to assist in decreasing cognitive decline [18, 23, 30], social factors did not explain the racial/ethnic differences in cognitive decline that were identified for progression to either MCI or dementia. This finding suggests that social participation and networking may not be the ideal target for interventions designed to mitigate racial/ethnic disparities in diabetes-related cognitive decline.

Clinically, greater awareness is needed regarding the importance of minimizing the cognitive complications of diabetes, particularly in racial/ethnic minorities. When providing information to older adults and caretakers on the importance of regular diabetes care to minimize the impact of complications, it may be important to highlight cognitive decline as an often-overlooked complication. In addition, incorporating routine assessment of cognitive function status during regular diabetes follow-up visits to find early signs of cognitive decline may improve clinical treatment [31]. Future research should investigate additional factors that may help explain disparities not available in this dataset, such as discrimination or disease specific measures of social support, and investigate social factors expected to have a positive influence and social factors expected to have a negative influence separately to understand if facilitating positive influences or mitigating negative influences may have a greater impact. It is important to note that variables included in this study are focused at the individual level, and macro-level contextual factors, such as structural racism, need further investigation to understand the full context in which racial/ethnic disparities in cognitive decline develop. Future studies should also include longer follow-up times, including measurements that begin earlier in life (such as educational quality) that may influence cognitive function later in life, and multiple measures of clinically important laboratory variables, such as hemoglobin A1C, depression, and physical activity to understand if changes in these clinical and lifestyle factors over time may explain racial/ethnic disparities in cognitive decline [32–34] and provide targets for intervention development. From a policy standpoint, regularly providing more culturally tailored and appropriate educational materials and resources for minority populations is important to ensure all racial/ethnic groups receive information to help manage diabetes and recognize the signs of cognitive decline [1]. Efforts are needed from a clinical, research, and policy standpoint to ensure current standards of care are maintained across racial/ethnic groups, and to find innovative programs that eliminate the disparities in diabetes across multiple complications, including cognitive decline.

Strengths of this study include the longitudinal, nationally representative dataset, the large sample size, the comprehensive set of possible explanatory variables, and the use of three different measures of cognitive function. However, there are limitations worth noting. First, while the data are longitudinal, the follow-up time period is limited. Further work is needed to understand how differences by race/ethnicity change over longer follow-up time. Second, though the dataset had a robust number of measures, it did lack HbA1c measures for the entire sample, depression, physical activity, and other factors that may be important targets for future interventions. Future studies that use datasets that can incorporate these factors, or that investigate specific relationships for mediation or moderation effects, or geographic variations in cognitive decline will provide further detail on intervention development. Finally, race/ethnicity as a social construct is known to be a proxy for several other social factors. Use as a crude measure to understand health disparities is necessary, but further work also needed to capture cultural and behavioral factors that underly self-reported race/ethnicity and may help identify drivers of disparities.

In conclusion, after adjusting for demographics, clinical/behavioral, and social factors, our results indicate that racial/ethnic disparities in cognitive decline amongst older adults with diabetes persist. Inclusion of mild cognitive impairment as an intermediate outcome measure between normal cognition and dementia allowed for a more comprehensive look at disparities across the spectrum of cognition, and recognition of the importance of diabetes management and provision of quality care across racial/ethnic groups early in progression to help prevent and support early recognition of signs of cognitive decline. Therefore, efforts should focus on preventive efforts such as increasing awareness of cognitive decline as an important complication of diabetes and increasing cognitive screening in older adults with diabetes especially ethnic minorities. In addition, this study found that sociodemographic, clinical/behavioral, and social factors did not explain the relationship between race/ethnicity and cognitive decline, and that more research is needed to understand the drivers of these differences by race/ethnicity.

## Data Availability

Data and materials will be made available by LEE upon request.
